# Nuciferine Prevents Hepatic Steatosis by Regulating Lipid Metabolismin Diabetic Rat Model

**DOI:** 10.1515/biol-2019-0079

**Published:** 2019-12-31

**Authors:** Qian Ning, Yang Wang, Yi Zhang, Guozhi Shen, Zhenglu Xie, Jie Pang

**Affiliations:** 1 Jinshan College of Fujian Agriculture and Forestry University, Fujian Province, 350001, P. R. China; 2 College of Food Science, Fujian Agriculture and Forestry University, Fujian Province, 350001, P. R. China; 3 Collaborative Innovation Center of Animal Health and Food Safety Application Technology in Fujian, Fujian Vocational College of Agriculture, Fuzhou City, Fujian Province, 350002, P.R. China

**Keywords:** Nuciferine, diabetic, rat model, hepatic steatosis, lipid metabolism

## Abstract

**Objective:**

This study investigatesthe nuciferine capacity to regulate the liver’s lipid metabolism regarding steatosis and injury in STZ-induced diabetic rats.

**Materials and Methods:**

The rats were randomly divided into groups control, diabetic and nuciferine 200 mg/kg/ day treatment. After 4 days of STZ injection, the nuciferine group was treated and administered via oral gavages for 4 weeks. At the end of experiment, blood, liver, myocardial and muscular samples were collected.

**Results:**

Nuciferine-treated significantly increased the body weight from 339.4g to 367.8g, but significantly decreased the food and water intake compared with diabetic rats. Also, the nuciferine-treated rats had significantly decreased TC, TG, and FFAs in the liver compared with the diabetic group, especially the serum markers of blood glucose. These were associated with the gene expression related to lipogenesis which was significantly down-regulated; the gene expression involved in lipolysis and fatty acid β-oxidation was significantly up-regulated. Discussion and

**Conclusion:**

The data provide evidence that nuciferine supplementation could protect the liver by regulating lipid metabolism gene expression resulting in decreasing the steatosis and injury in diabetic rat. Thus, nuciferine could be developed as a diabetic adjuvant food additive in future.

## 1 Introduction

Previous studies have shown that alkaloids have anti-diabetic effects involving endocrine metabolic disorders characterized by chronic inflammation, which can consequently cause long-term dysfunctions in the liver [1, 2, 3, 4, 5]. Nuciferine also has confirmed anti-diabetic effects by *in vivo* studies [6, 7]. The leaves of *Nelumbo nucifera* are a medicinal herb showing great effects in China, which are recorded in a documentation of traditional Chinese medicine [8, 9]. Nuciferine is a major aporphine alkaloid from *Nelumbo nucifera* and is a medicinal herb used in the process of sunstroke, dysentery, and blood clotting [10], anti-obesity [11], hypolipidemic [12], and anti-diabetic [13]. With regard to the dyslipidemia, previous studies have showed that nuciferine has proved to have value in terms of ameliorated liver steatosis and injury in the high-fat diet induced rat model [12]. Additionally, the nuciferine supplementation could change the gene expression of lipid metabolism in hepatic including SREBP-1c, acetyl carboxylase (ACC), fatty acid synthetase (FAS), carnitine palmitoyl transferase (CPT-1) and PPAR-α [14, 15].

The various physiological activities of nuciferine have been demonstrated, but whether these links to liver lipid metabolism in the DM rat model is not yet clear. Therefore, this study aimed to investigate the nuciferine capacity to regulate liver lipid metabolism about the steatosis and injury in STZ-induced diabetic rats. These findings provide a novel insight into the protecting effect of nuciferine associated with the changing of hepatic gene expression involved in lipid metabolism by nuciferine supplementation.

## 2 Material and methods

### 2.1 Animals and ethics statement

Forty-five six-week-old Sprague-Dawley (SD, Male) rats (205±3.9 g) were obtained from Shanghai SLAC Laboratory Animal CO. LTD (Shanghai, China). All animals were acclimatized to the laboratory conditions for one week, then weighed and given a random allocation in this study.

**Ethical approval**: The research related to animals use has been complied with all the relevant national regulations and institutional policies for the care and use of animals. All experimental procedures were referred to the care of laboratory animals of the Fujian province Zoological Society and approved by the Animal Care of Fujian Agriculture and Forestry University (Permit No. PZCASFAFU2018005).

### 2.2 Design and sampling

The rats were fed under a controlled temperature of 22±3°C and a 12h light/dark cycle. Standard food pellets and tap water were used during the experimental process. After acclimatization to the experimental environment for one week, the rat’s diabetes model was induced by intraperitoneal (i.p.) injection a dose of 70 mg/ kg of STZ (Sigma, USA), which was diluted in citrate buffer (20 mM, pH 4.5). Three-days later, blood glucose of tail vein sampling was measured by Medisafe chips (Terumo, Japan). The morning blood glucose concentration of 300 mg/dL was defined as the diabetes model. The rats were randomly allocated into three groups, each group contained 15 rats. They included the control group (NC) and diabetic rat group which contained the 0.9% normal saline treated group (PC) and treated with nuciferine 200 mg/kg/day group (Nuci). The nuciferine was purchased from Macklin (Shanghai, China) and diluted in 0.9% normal saline. After 4 days of STZ injections, the Nuci group was treated and administered via oral gavages with nuciferine for 4 weeks. During the experiment, the body weights and blood glucose levels were recorded every week. After treatment, the animals were fasted for 12 h and then the fasting blood glucose levels of all rats were measured and considered diabetic when the levels exceeded 11.1 mmol/l. The intakes of food and water were recorded every day. The water intake was measured by the weight of the bottle. At the end of the experiment, the rats were given a mild ether anesthesia and a blood sample was collected in a heparin sodium vessel. Subsequently, the plasma was collected after centrifugation for 15 min in 3000 rpm and stored at -20 °C until we analyzed the lipid level. After the blood collection, liver, myocardial and muscular samples were collected by sacrificing the rat. The liver samples were divided into three parts. 1) Liver tissues were immediately collected, weighted, and a part was stored at-80 °C until the gene expression was analyzed. 2) A part of the liver was stored at -20 °C until the lipid level was analyzed. 3) 1 cm^3^ tissue was stored at 4% paraformaldehyde until histopathological analysis.

### 2.3 Biochemistry analytical

The plasma triglyceride (TG), total cholesterol (TC) and glucose levels were measured using commercial kits (Tiangen, Nanjing, China) by enzymatic methods. The plasma free fatty acids (FFAs) concentration was measured using commercial kits and according to the specification strictly (Wako, Japan) by ACS-ACOD method. Plasma levels of low-density lipoprotein (LDL) and high-density lipoprotein (HDL) were measured according to the commercial Elisa kit manufacturer’s protocol (Langdun Technologies Inc, Shanghai, China).

### 2.4 Tissue glycogen content determination

Glycogen contents of the myocardial, hepaticand muscular were measured with commercial kits (Tiangen, Nanjing, China) and normalized to protein levels (Beyotime, Nantong, China).

### 2.5 Histopathological analysis

The paraformaldehyde-fixed livers were stained with the H.E. Methodor Periodic Acid Schiff. Morphological analysis was analyzed microscopic fields per section by computerized image analysis system.

### 2.6 RT-PCR analysis

Total RNA was extracted from the liver tissue according to the TRIzol kits manufacturer’s protocol (TaKaRa, Dalian, China). The RNA concentration was quantified by spectrophotometer (Eppendorf-Biotech, Hamburg, Germany). The RNA samples (2 mg) were reverse transcribed according to the manufacturer’s instructions of the cDNA synthesis kit which was purchased from Applied Biosystems Co. Ltd. (UK). The RT-PCR was performed to analyse the gene expression by the MyiQ2 Real-time PCR system (Bio-Rad, Hercules, USA). PCR reaction used 5μM primers, 10 ng sample cDNA, and 10μL SYBR Mix. PCR reaction conditions include an initial denaturing cycle at 95 oC for 5 min, followed by 40 amplification cycles: 15 s at 95 oC and 45 s at 60 oC. The primer (Table 1) of a sterol regulatory element binding protein 1c (SREBP-1c) and liver X receptor-α (LXR-α) was used from the previous study [16, 17]. The gene expression was analyzed according to the previous study [18, 19].

### 2.7 Statistical analysis

The results were expressed as mean±SEM and the statistical significance was evaluated using the SPSS-20.0 software by LSD modelin ANOVA. P < 0.05 was considered to indicate statistical significance.

## 3 Results

### 3.1 BW, food and water intake

**Figure 1:**
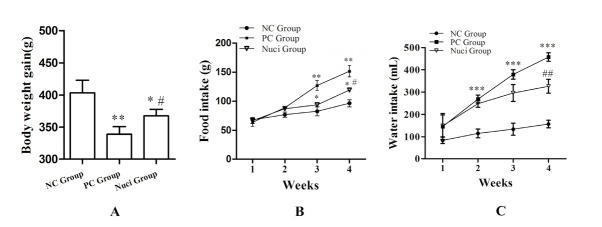
**Body weight, food intake, and water intake**. A, the body weight of rats; B, the food intake; C, the water intake. Data are presented as themeans ± SEM of determinations (n =15), indicates statistically significant differences when compared with NC group, * P<0.05 and ** P<0.01; indicates statistically significant differences when compared with PC group, # P<0.05 and ## P<0.01.NC was a control group and the rats were allowed to free access to a normal diet; PC was a diabetic control group; Nuci was a treated group and the diabetic animals were treated with nuciferine 200 mg/kg/day, below the abbreviation of the same.

In this experiment, the BW of rats was obviously decreased in the PC group and Nuci-group compared to those in the NC group (Figure 1A). However, the BW of rats was significantly increased in the Nuci-group (P<0.05) compared with the PC group (Figure 1A). During the experiment, the food intake in the PC group and in the Nuci-group was higher than the NC group. Importantly, they were significantly increased in the PC group (P<0.01) and in the Nuci-group (P<0.05) by the 3rd week when compared with the NC group (Figure 1B), but the food intake showed a significant decrease in the Nuci-groups at the 4th week compared with the PC group (Figure 1B). The variation trend of the water intake was the same as food intake and shown in Figure 1C.

### 3.2 Plasma and liver lipid profiles

As shown in Table 2, the concentration of plasma TG, TC, glucose, FFAs and LDL was significantly increased in the PC group compared with the NC group (P<0.05), while HDL concentration was markedly decreased (P<0.001). The plasma glucose, TG, FFAs, TC and LDL concentrations were significantly decreased (P<0.05) when the rats were treated with nuciferine. Although the plasma glucose was still higher in the Nuci-group, the nuciferine treated rats showed significantly decreased glucose levels (P<0.05). The plasma HDL concentration was also significantly increased in the Nuci-group compared with the PC group (P<0.05). The contents of TC, TG, and FFA in liver were significantly increased in the PC group compared with the NC group (P<0.05), but comparing with the nuciferine supplementation, this effect was significantly reversed with (P<0.05).

### 3.3 Tissue glycogen content

As Table 3 shows, the myocardial (P<0.05), hepatic (P<0.01) and muscular (P<0.05) glycogen concentration in the PC group was significant lower than of that in the NC group. Nuciferine treated rats showed obviously increased myocardial, hepatic and muscular glycogen concentrations compared with the PC group (P<0.05), but the hepatic and muscular glycogen levels were obvious decreased compared with the NC group (P<0.05).

### 3.4 Histological analysis

**Figure 2:**
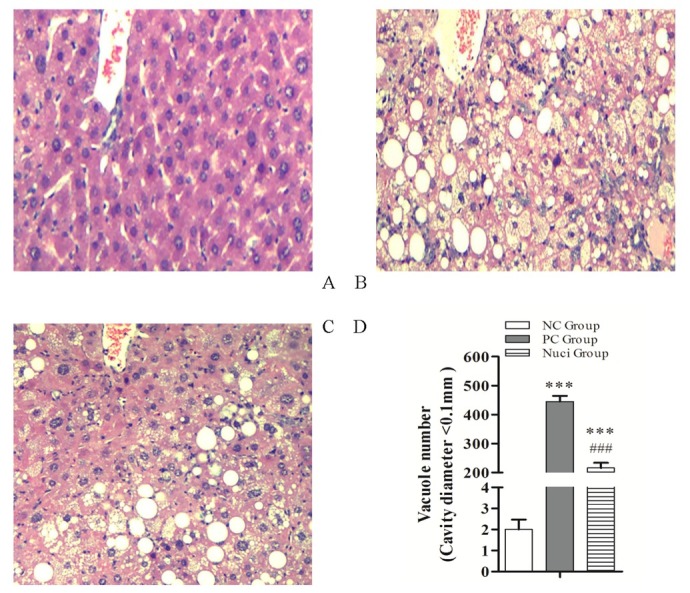
**Histological examination of the liver tissue (10×) of SD rats following HE staining**. (A) Normal control diet group; (B) diabetic rats treated with 0.9% normal saline; (C) diabetic rats treated with nuciferine which used 200 mg/kg/day diluted in 0.9% normal saline; (D) Vacuole number. About the counts for the fat vacuole, which was required the diameter of the cavity is less than 0.1 mm. The data of vacuole number are presented as the means ± SEM of determinations, indicates statistically significant differences when compared with NC group, * P<0.05 and *** P<0.001; indicates statistically significant differences when compared with PC group, # P<0.05 and ### P<0.001.

Hepatic histological examination showeda central vein located in the center of hepatic lobe, a central vein that is radiating and arranging by hepatic cord or plate. The hepatic cords or masses are irregular in the livers of the NC group (Figure 2A). By contrast, the liver cells showed serious stromal inflammatory cell infiltration and fatty degeneration. Meanwhile, abundant and large lipid droplets within hepatocytes and lipid vacuoles appeared in the cytoplasm (Figure 2B). The fat cells’ volume and size in the PC group were larger than the NC group. However, liver cell degeneration was lessened when the rats were fed with nuciferine for 4 weeks (Figure 2C). Importantly, the volume and the size of fat cells were shrunk in the Nuci-group compared with those of the PC group. If the diameter of the cavity was less than 0.1 mm, it was recorded. The vacuole numbers were significantly decreased (P<0.001) in the Nuci-group compared with the PC group (Figure 2D).

### 3.5 Gene expression of lipid metabolism

**Figure 3:**
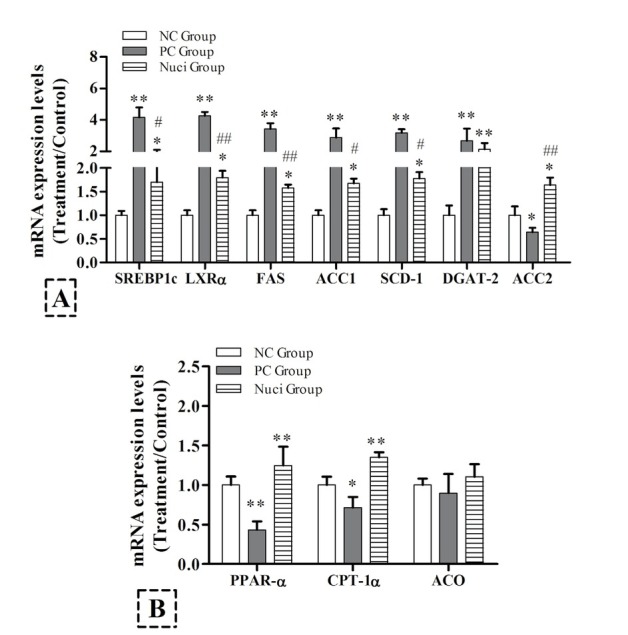
**Effects of nuciferine on hepatic mRNA expressioninvolved in lipid metabolism**. RNA was extracted from the liver andreverse transcribed to cDNA and analyzed by qRT-RCR for gene expression. The experiments were used 8 samples from each group. Data are presented as the means ± SEM, indicates statistically significant differences when compared with NC group, * P<0.05, ** P<0.01 and *** P<0.001; indicates statistically significant differences when compared with PC group, # P<0.05, ## P<0.01, ### P<0.001.

The effects of nuciferine on the gene expressions of lipid metabolism in the liver tissues of SD rats were measured by RT-PCR. As shown in Figure 3, gene expression levels of SREBP-1c, LXR-a, ACC1, SCD-1, FAS, and DGAT-2 were significantly upregulated in the PC group compared with those in the NC group, but these genes expression were significantly inhibited by dietary nuciferine supplementation except DGAT-2 (P<0.05; Figure 3A). Importantly, the ACC2 was significantly decreased in the PC group, but dietary nuciferine supplementation reversed this effect (P<0.05). With the fatty acid β-oxidation, PPAR-α and CPT-1α gene expression levels were significantly decreased in the PC group compared with the NC group (P<0.05). Compared with the PC group, PPAR-α and CPT-1α gene expression levels were significantly upregulated with the nuciferine supplementation (P<0.05). However, the acyl-CoA oxidase (ACO) expression level did not differ among groups (Figure 3B).

## 4 Discussion

This study demonstrates that the effect of nuciferine alleviated the liver injury by regulating lipid metabolism used diabetes models by STZ [2, 3, 4, 20]. As shown inpresent results, the blood glucose level was markedly elevated more than three-fold in the STZ-treated rats compared with the NC group, whereas the blood glucose level was attenuated significantly in the nuciferine supplemented (Table2). Some studies showed that dietary supplements for nuciferine have an effect to control glycemic [6, 13, 21]. At the same time, nuciferine treated diabetic animals significantly increased their hepatic and myocardial glycogen levels compared with diabetic animals in the PC group (Table 3). These results imply that nuciferine could prevent diabetic pathological conditions by reducing hyperglycemia.

However, during diabetic conditions, liver steatosis was induced in the diabetic rat model with dyslipidemia (Figure 2). According the present study, this model is a good choice to analyse metabolic syndrome [22, 23, 24]. In this study, the plasma TC, TG, FFA and LDL-C concentrations, and the liver TC, TG and FFA concentrations were significantly increased in the diabetic rats. However, nuciferine supplementation could attenuate the plasma and liver TC, TG, and FFA contents. In addition, the results of microscopic analysis have shown that nuciferine supplementation also attenuated the hepatic steatosis. These data demonstrated that nuciferine could prevent steatosis development in the liver by regulation of the plasma lipid profile.

As previously studied, the liver fat accumulation is involved in the process of metabolic syndrome, which was associated with the cluster of metabolic abnormalities [25]. Generally, intracellular lipotoxic injury was induced by the abnormal metabolism of FFAs and their derivatives [26]. And besides, high increases in lipid droplet content is an important hallmark of developing diabetes mellitus [27, 28, 29]. Our results were also showed that hyperlipidemic mice fed with nuciferine, decreasing the FFAs concentrations, and indicated theminimal hepatic steatosis.

During the process of chronic hyperinsulinemia, lipogenic transcription factors LXR-α was upregulated resulting in hepatic lipogenesis increased [30]. Previous studies have shown that LXR-α could enhance lipogenesis gene transcription SREBP-1c [31, 32, 33]. Especially, activating SREBP-1 to regulate the lipogenic enzymes of ACC1 and FAS to inhibit lipogenesis [31, 33, 34]. In our study, the SREBP-1 expression was up-regulated in the PC group; by contrast, dietary nuciferine supplementation reversed this effect. Meanwhile, dietary nuciferine supplementation also significantly reduced the gene expressions of FAS and ACC1, but significantly increased the ACC2 expression. This is consistent with the previous study and descript that ACC2 regulates fatty acid oxidation whereas ACC1 maintains regulation of fatty acid synthesis [35, 36]. According to the previous result, the SREBP-1c gene could activate by LXRα, which is a form with retinoid X receptors [37, 38]. Importantly, LXRα can bind the promoter of SREBP-1c and induce FAS activated [38]. In the present study, the expression level of LXRα gene was down-regulated with dietary nuciferine supplementation compared with the PC group and consistent with previous research. Additionally, the previous research showed that increased SREBP activity is increased by activation of PPARα [39, 40]. Our results show that the gene expression of PPARα in the Nuci-group compared with the PC group. These results suggest that PPARα activation could be enhanced by the SREBP pathway resulting in hepatic lipid accumulation decreased and inflammatory responses attenuated. Therefore, we postulate that dietary nuciferine supplementation could improve liver steatosis by decreased expression of lipogenesis gene. Importantly, the gene expression of CPT-1α and ACO was significantly increased in the Nuci-group. They are major enzymes responsive to PPARα activation to upregulate the process of fatty acid β-oxidation [39]. Thus, the beneficial effects of nuciferine could control the hepatic lipid metabolism disorders by regulating lipids β-oxidation in the liver.

## 5 Conclusions

In conclusion, we propose that a protective effect of nuciferine on liver steatosis and injury in the STZ-induced diabetic model. The result indicated that nuciferine could be likely regulating the gene expression related to liver lipid metabolism.

**Author contributions**: QN, YW, YZ and GS performed the experiment, analyzed the data, contributed reagents/ materials/analysis tools, wrote and approved the manuscript. ZX and JP conceived and designed the experiments, revised and approved final manuscript.
